# Supplementary material for: Spatial distribution of stunting among breast feeding children in Sub-Sahara Africa
Source: PLoS One. 2025 Jun 13;20(6):e0325812. doi: 10.1371/journal.pone.0325812 (PMC12165360; doi:10.1371/journal.pone.0325812)
Supplement: Attachment — Submitted filename: Peer Review Report- Breast Feeding.docx [file pone.0325812.s001.docx]

**Peer Review Report**

**Title:** Spatial distribution of stunting among breast feeding children in Sub-Sahara Africa
**Authors:** Bekahegn Girma^1*,^ Lealem Dibku Sasahu^2^ and Azizur Rahman^3^

**Reviewer:** Luqman Abass
**Date:** 2/13/2025

**Originality and Novelty**

The study presents original research on the spatial distribution of stunting among breastfeeding children in Sub-Saharan Africa (SSA). While previous studies have examined stunting in specific countries, this research provides a comprehensive analysis across 31 SSA nations, filling a gap in the literature.

**Methodology and Technical Standards**

The researchers employed a robust methodology using Demographic and Health Survey (DHS) data from 31 SSA countries, encompassing 174,586 breastfeeding children across 15,398 clusters. The use of Geographic Information Systems (GIS) and spatial analytic methods, such as Getis-Ord Gi* and Global Moran's I, demonstrates a high technical standard in analyzing spatial patterns of stunting.

**Data Analysis and Presentation**

The statistical analysis appears to be conducted rigorously, with appropriate weighting to account for varying response rates and sampling probabilities. The use of spatial autocorrelation techniques and interpolation methods provides a comprehensive view of stunting distribution across SSA.

**Results and Conclusions**

The study's findings are presented clearly, with stunting prevalence ranging from 17.63% to 53.68% across different SSA countries. The identification of hotspots in Central and Eastern Africa, and cold spots in Southern Africa, is well-supported by the data and analysis presented. The conclusions drawn from these results appear appropriate and align with the study's objectives.

**Clarity and Language**

The manuscript is written in standard English and is generally intelligible. However, there are some minor grammatical errors and awkward phrasings that could be improved for clarity.

**Ethical Considerations**

While not explicitly stated in the provided excerpt, the use of DHS data typically adheres to ethical standards. However, the manuscript should include a statement on ethical approval and data usage permissions to ensure full compliance with research integrity standards.

**Reporting Guidelines and Data Availability**

The study appears to follow appropriate reporting guidelines for spatial analysis research. However, a statement on data availability should be included to meet community standards.

**Areas for Improvement**

1. Literature Review: The background section could benefit from a more comprehensive review of recent literature on stunting in SSA, particularly studies using similar spatial analysis techniques.

2. Discussion: Expand on the implications of the findings for public health policy and interventions in SSA.

3. Limitations: Include a dedicated section discussing the study's limitations, such as potential biases in DHS data or challenges in cross-country comparisons.
